# Phase 1 Trial of Amnion Cell Therapy for Ischemic Stroke

**DOI:** 10.3389/fneur.2018.00198

**Published:** 2018-06-07

**Authors:** Thanh G. Phan, Henry Ma, Rebecca Lim, Christopher G. Sobey, Euan M. Wallace

**Affiliations:** ^1^Clinical Trials, Imaging and Informatics (CTI) Division, Stroke & Ageing Research (STARC), Department of Medicine, School of Clinical Sciences at Monash Health, Monash University, Clayton, VIC, Australia; ^2^The Ritchie Centre, Department of Obstetrics and Gynaecology, Hudson Institute of Medical Research, Monash Health and Monash University, Melbourne, VIC, Australia; ^3^Department of Physiology, Anatomy and Microbiology, School of Life Sciences, LaTrobe University, Melbourne, VIC, Australia

**Keywords:** stroke, stem cell, amnion, phase 1, trial

## Abstract

**Background:**

There is increasing interest in stem cell therapy as another treatment modality in stroke, particularly for patients who are unable to receive endovascular clot retrieval or thrombolysis therapies, or for whom standard treatment has failed. We have recently shown that human amniotic epithelial cells (hAECs) are effective in reducing infarct volume in different animal models of ischemic stroke, including in non-human primates. hAEC therapy attenuated infarct growth and/or promoted functional recovery, even when administered 1–3 days after the onset of stroke.

**Methods:**

We now propose an open label Phase 1 dose escalation trial to assess the safety of allogeneic hAECs in stroke patients with a view to providing an evidence platform for future Phase 2 efficacy trials. We propose a modified 3 + 3 dose escalation study design with additional components for measuring magnetic resonance signal of efficacy as well as the effect of hAECs on immunosuppression after stroke.

**Result:**

The trial will commence in 2018. The findings will be published in a peer-reviewed journal.

**Conclusion:**

The trial is registered with ANZCTR (ACTRN12618000076279p).

## Introduction

Prior to 2015, the standard clinical approach for reducing disability and death in ischemic stroke was Stroke Unit admission, antiplatelet therapy, and treatment with recombinant tissue plasminogen activator (TPA), within 4.5 h from onset in selected patients (1). However, the rate of vessel re-opening when there is a large obstructive clot is low (12%), even with TPA (2). Since 2015, there has been considerable optimism about the place of endovascular clot retrieval (ECR), for large clot removal, as a new and transformative therapy (1). However, even with this extended time window (up to 24 h), the number of eligible patients for ECR is less than 15% (1, 3). Further, a substantial proportion of those patients in the trial who received these treatments was left with moderate to severe disability or died (72% of patients who received TPA and 48% of those who received TPA and ECR) (2). In short, there remain enormous challenges in achieving the effective management of stroke across all patients. There is thus an urgent need to explore alternative therapeutic strategies that may be both suitable for more stroke patients and be more effective in improving outcome after stroke.

One such strategy is to move from the current time-based approach to an imaging tissue-based approach where treatment is based on the presence of salvageable ischemic tissue (3, 4). However, reperfusion therapies shift the peri-infarct zone toward the ischemic core but do not address the local and systemic immune response cascades that separate from the ischemic insult itself, promoting further tissue damage surrounding the infarct (5). The body’s intended effect of post-stroke immunosuppression appears to be the dampening of the “autoaggressive” Th1 immune response within the brain (6), but the unintended consequence is an increased risk of infection such as pneumonia (7), and this phenomenon is associated with high mortality and poor functional outcomes. These infiltrating inflammatory cells are located in the peri-infarct area, which has been referred to as the “inflammatory penumbra,” which denotes an area susceptible to further injury from the inflammatory cascade (5). Recognition of the need to manage the inflammatory response as part of stroke therapy has led to a call for future therapies that also focus on mitigating injurious events in the peri-infarct zone and not just on reperfusion therapy (5). In this regard, stem cell therapy has emerged as one potentially attractive option because of its ability to modulate inflammatory pathways at multiple sites appropriate to the changing pathophysiological state over time (8). By contrast, an example of a unimodal neuroprotectant drug target that would be unsuitable for modulating the inflammatory cascade is matrix metalloproteinase (MMP-9). In the early phase of stroke, MMP-9 damages the blood–brain barrier (BBB) (9) and contributes to vasogenic edema, whereas in later stages, it contributes to remodeling around the peri-infarct areas, and consequently targeting MMP-9 at this time can be harmful (5).

### Human Amnion Epithelial Cell as a Cell Therapy

**Figure 1:**
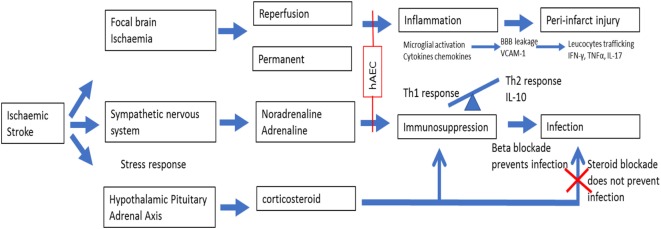
Action of amnion stem cell on inflammatory cascade post ischemic stroke.

The amniotic membrane is the inner layer of the fetal sac and consists of epithelium, basement membrane, and stroma. Amnion epithelial cells are therefore adult cells that express surface markers common to those of embryonic stem cells, primordial germ cells, and somatic stem cells including neural crest cells (10). Human amniotic epithelial cells (hAECs) express nonpolymorphic, nonclassical human leukocyte antigen (HLA) G (11) and have “low” immunogenicity (12) due to their role in protecting the fetus from immune rejection. There are various mechanisms by which hAECs might exert therapeutic effects following stroke. First, hAECs could secrete neurotrophic factors that promote the recovery of damaged cells in the penumbra. Such factors could also promote synaptogenesis to re-innervate lost connections. Second, hAECs could differentiate into a neuronal phenotype and replace damaged or dead cells. Third, hAECs could act as “biological minipumps” within the central nervous system, secreting necessary cytokines, growth factors, hormones, and/or neurotransmitters to restore cellular function. Fourth, hAECs could improve stroke outcome by dampening of the innate and adaptive immune systems (suppression of pro-inflammatory cytokines and secretion of factors that inhibit chemotactic activity of neutrophils and macrophages) that contributes to brain injury, which would include protection of neurons from immune cell-mediated apoptosis (10, 13). Lastly, systemically administered hAECs could attenuate the severity of post-stroke immunosuppression involving marked leukopenia and atrophy of secondary lymphoid tissues. Figure 1 provides a schematic of the potential effects of hAECs on post-stroke inflammation and immunosuppression.

We have evaluated the utility of an intravenous route for hAEC therapy in a series of experiments involving different animal models of stroke (C57Bl6 mice and marmoset monkeys; both sexes) (14). These experiments showed that hAECs can reduce infarct volume and functional deficit when given acutely or as long as 3 days after stroke onset (14). hAECs were detected in the brain (infarct) and spleen of animals subjected to stroke, but not in sham-operated animals that received hAEC (14), indicating that hAEC migrate to tissues impacted by stroke. Key effects at the peri-infarct zone and infarct core occurred *via* reductions in (1) apoptosis, (2) inflammation (i.e., reduced expression of inflammasome protein, NLRP3 at 24 h and dampened entry of neutrophils, CD8^+^ T cells, B cells, and pro-inflammatory macrophages into the infarct by 72 h), and (3) post-stroke immunosuppression (14). Over 8 weeks, there was a decreased spread of gliosis into the adjacent border in hAEC-treated mice. Further, long-term functional recovery was still augmented in either young or aged mice of both sexes even if hAEC administration was delayed until 1 or 3 days post stroke (14).

## Method

### Overall Plan

We have designed an open label Phase 1 dose escalation trial of hAECs in patients with ischemic stroke with the primary aim of determining the maximal tolerated dose (MTD). Assessment of adverse events will be undertaken blinded to cell dose. Fifteen patients will be recruited for a dose escalation trial in 2018. The final follow-up for the 15th patient in the hAEC arm will be done 12 months after cell delivery. The trial will be submitted to the Monash Health Human Research Ethics Committee.

### Inclusion Criteria

Patients are eligible if (1) they have ischemic stroke in the territory of the large main artery (middle cerebral artery) (15), (2) present within 24 h of stroke onset and are not eligible for TPA or clot retrieval, (3) age is between 18 and 85 years old, (4) have National Institute of Health Stroke Scale/NIHSS (tool used in clinical trials for measuring stroke severity) between 6 and 15, and (5) patient or person responsible (on behalf of participant) must sign consent form after explanation of the trial.

### Exclusion Criteria

Patients are excluded if (1) eligible for TPA and/or ECR; (2) there is evidence of autoimmune disease, organ transplant, malignancy, splenectomized individuals, or have infection at the time of stroke; (3) neurodegenerative disease such as dementia or Parkinson’s disease; (4) pregnancy; (5) have contraindications for magnetic resonance (MR) imaging such as pacemaker; (6) patients with initial infarct [on diffusion weighted imaging (DWI)] volume of ≤5 or >100 mL will also be excluded (14, 16). This is a common strategy used in many studies to exclude infarcts with very small volume (such as lacunar infarct); very large infarcts are excluded as these patients have a high probability of developing malignant middle cerebral artery infarct and death; (7) patients with mild stroke (NIHSS <6) or very severe stroke (NIHSS >15); (8) blood sugar level of ≤3 or ≥20 mmol/L; and (9) significant lung disease requiring oxygen.

### Treatment Doses

Treatment doses are of stage-wise dose escalation: 2, 4, 8, 16, and 32 million cells/kg.

### Infusion Protocol

Patients will be given hAECs by intravenous infusion for 1 h, previously shown to be safe in preterm babies. A recent study with multipotent adult progenitor cells showed that 1,200 million cells were infused up for 1 h (17). The maximal dose used in neural stem cell trial was 20 million cells/kg given by direct intracerebral injection (18).

### Sample Size

**Figure 2:**
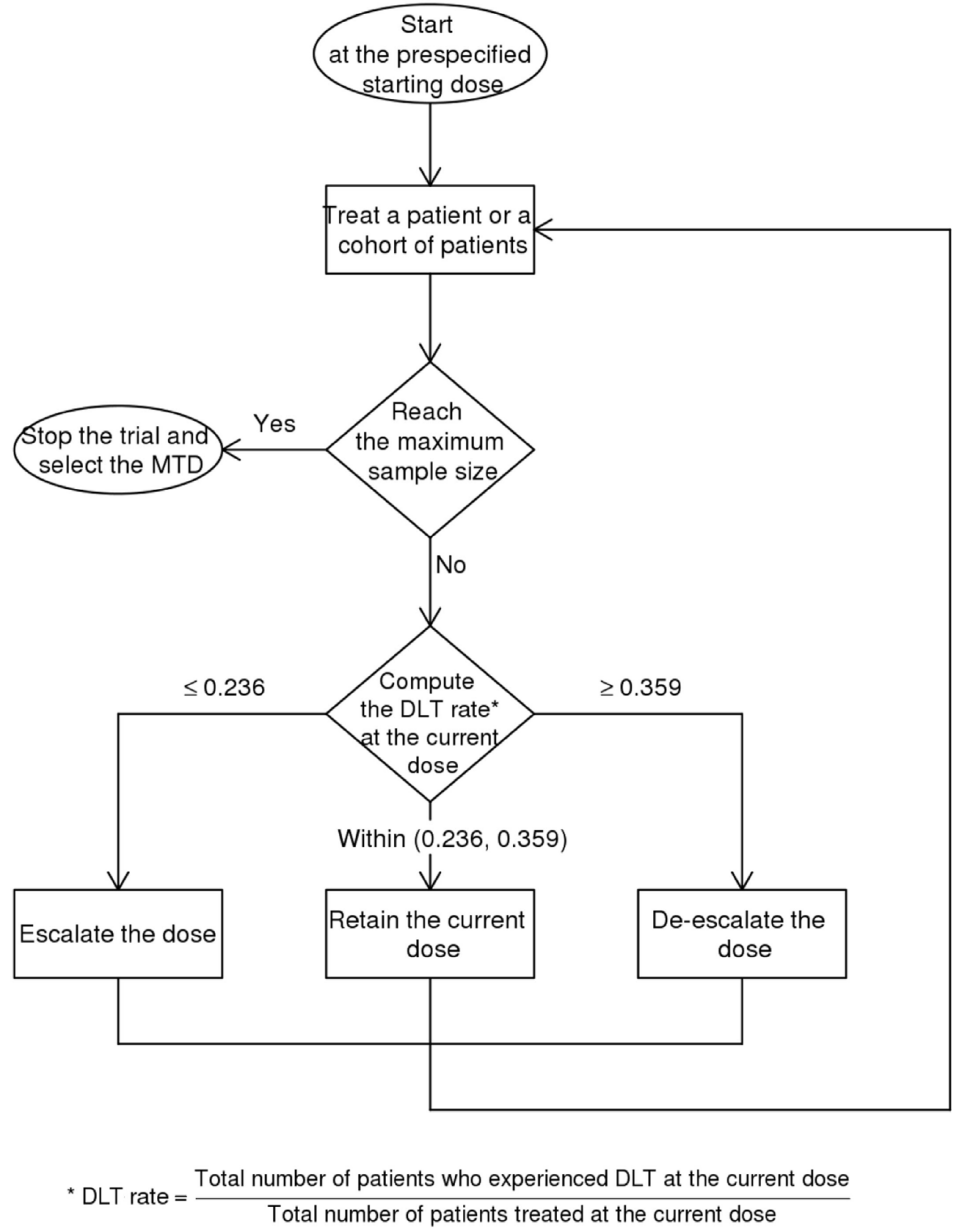
Schematics of dose escalation and de-escalation.

This trial is designed as a balance between finding the MTD and finding the effective dose (19). The dose escalation and de-escalation stages are performed similar to the classic 3 + 3 scheme in a Phase 1 trial whereby the sample size is increased in small increments of 3. For a 5-dose escalation, the estimated sample size is 15. The Bayesian Optimal Interval Design (BOIN) (20) used here weighs the dose escalation by the lower boundary of the target toxicity rate (safety rate = 0.6 × target) and de-escalation by the upper boundary (toxicity rate = 1.4 × target). This method recommends setting the default target rate at 0.3 rather than a lower value because of the small sample size typical for Phase 1 trial (20). This default is higher than 90-day mortality of 15.7% for patients at the Monash Medical Centre with NIHSS between 6 and 15. The map of this approach is provided in Figure 2 and Table 1. The rows show that the decision for escalating or de-escalating the dose can be performed by counting the number of patients with the observed toxicity.

**Table 1:** Schematics of escalation and de-escalation dose [dose-limiting toxicity (DLT)].

Human amniotic epithelial cell dose (million cells/kg)	2	2	2	4	4	4	8	8	8	16	16	16	32	32	32
Patient number	1	2	3	4	5	6	7	8	9	10	11	12	13	14	15
Escalate drug dose if number of patients with DLT is ≤	0	0	0	0	1	1	1	1	2	2	2	2	3	3	3
De-escalate drug dose if number of patients with DLT is ≥	1	1	2	2	2	3	3	3	4	4	4	5	5	6	6
Stop trial if number of patients with DLT is ≥	NA	NA	3	3	4	4	5	5	5	6	6	7	7	8	8

### Statistical Analysis

Descriptive statistics will be used to describe patient demographics, safety, and SAE of hAECs. The dose–response curve for estimating the MTD is performed using the BOIN package (20). This method computes the probability of toxicity (>0.3) given data base on the list of the number of patients treated at each dose and the number of toxicity at each dose. The MTD is denoted by a marked rise in the probability of toxicity from baseline.

### Data and Safety Monitoring Board (DSMB)

The DSMB will be composed of stroke physicians with experience in clinical trials. Similar to other stem cell trial, after the last subject in cohort 1 (dose 2 million cells/kg) has been observed for 7 days after infusion, the DSMB will evaluate the dose-limiting toxicity (DLT) and other safety information for the eight subjects to determine whether it is safe to proceed to cohort 2.

### Human Amniotic Epithelial Cells

The Cell Therapies and Regenerative Medicine Platform (CTRMP) at the Monash Health Translation Precinct has a cryobank of isolated, screened, and stored hAECs for clinical trial use (21). These cells are routinely obtained from healthy women with a healthy singleton pregnancy undergoing an elective cesarean section at term and donating their placenta for clinical trial purposes with informed consent. The donor-screening process involves a two-page medical history questionnaire and viral serology testing. The hAECs are isolated in compliance with current Good Manufacturing Practices using the Biospherix Xvivo cell isolator located at the CTRMP. The manufacturing process is undertaken free of animal-based products, and the isolated cells are cryopreserved without culture expansion. An aliquot of the isolated cells is subjected to microbial and endotoxin testing as part of the cell release protocol (below).

The release criteria for the hAECs are as follows (22):

1. Absence of microbial contamination and endotoxins.
2. >96% epithelial cells—determined by flow cytometric analysis of EpCAM expression.
3. <1% mesenchymal stromal cells and leukocytes—determined by flow cytometric analysis of CD90, CD105, and CD45.
4. >80% post-thaw viability.
5. >50% reduction in T cell proliferation index.
6. >25% reduction in M1 (CD86^+^) macrophages and >10-fold increase in M2 (CD206^+^) macrophages—determined by macrophage polarization assay.

### Clinical Outcomes (Aim 1)

The primary clinical outcome is safety or frequency of SAEs. The secondary clinical outcome is the patients’ stroke severity (measured by NIHSS) and the level of disability (measured by modified Rankin scale) at 1 week and 90 days. The modified Rankin scale is a widely used tool for measuring outcome in stroke trials. A score of 0 indicates no disability and 6 indicates death. Neurological impairment and functional scores will be measured by a neurologist. The NIHSS will be assessed before the acute study, then again at 24 h, at time points coinciding with MRI scanning (1 week, 1 month, and 12 months), and at any other time during the hospital stay if there is a clinical deterioration. *The neurological and safety outcome assessment will therefore be performed by a blinded observer. This observer will not be involved in patient recruitment*.

### Safety

We define a treatment-emergent adverse effect/AE (TEAE) as any event not present before the initiation of treatment or any event already present that worsened in either intensity or frequency after exposure to the study treatment (23). We classify TEAEs as mild, moderate, severe, or life threatening according to standard procedures (23). DLT is defined here to be equivalent to SAE and includes any untoward effect such as death, life threatening, requires hospitalization (for outpatient), prolonged hospitalization (for inpatients), or requires intervention to prevent permanent damage.

### Imaging Outcomes

The primary imaging outcome is the volume of vasogenic edema at 1 week versus that before treatment. This analysis is performed with a view to assessing safety. The secondary imaging outcome is the change in the infarct expansion ratio (IER) between the outcome post-treatment (T2 FLAIR) infarct volume and the initial DWI infarct volume (see Image Analysis) (16), and symptomatic intracerebral hemorrhage on MR or CT (4). Symptomatic intracerebral hemorrhage is defined by Safe Implementation of Thrombolysis in Stroke Monitoring Study (SIT-MOST) criteria as parenchymal hematoma type 2 (PH2) occurring within 3–6 hours of treatment, combined with neurological deterioration leading to an increase of four points on the NIHSS from baseline, or the lowest NIHSS value after baseline to 24 h (24).

### Imaging Protocol

Patients will have MR imaging performed prior to receiving hAECs and at 1 week, 1 month, and at 12 months. The sequences include volumetric 3D T1 images, T2 weighted FLAIR images, DWI (measures infarct volume) and tensor imaging/DTI (assesses integrity of white matter tracts), MR angiography (assesses blood vessel patency), arterial spin labeling (assesses cerebral blood flow), and susceptibility weighted imaging (detection of blood products). The choice of these MR sequences is based on consensus recommendations from an expert panel: Stem Cells as an Emerging Paradigm in Stroke (STEPS) 3 (8).

### Image Analysis

Images will be de-identified prior to analysis. The analysis will therefore be performed blinded to the dosing regiments. Segmentation of infarct will be performed using in-house purpose-written semi-automated software. In a previous analysis from our group, the IER in the control arm occurred in 75% of patients (16). Hyperintense signal on FLAIR images consists of the infarct (cytotoxic edema) and surrounding vasogenic edema. By contrast, the lesion on DWI consists of cytotoxic but not vasogenic edema. Vasogenic edema is therefore defined as the difference between the hyperintense signal on FLAIR sequence at day 7 minus the hyperintense signal on the DWI sequence (all sequences will be co-registered to the same coordinate space for analysis). The DTI images will be used to characterize longitudinal change in the fractional anisotropy (white matter integrity) around the infarct. Used in this way, it will provide another measure of peri-infarct injury and/or recovery.

### Immunological Assay

We will perform studies to evaluate the effects of the hAECs on the immune response post stroke. Assessments will be made before hAEC administration, 1 h after hAEC infusion, and at 24 h, 3 days, and 1 week. Venepuncture will be performed using EDTA tubes at the time of enrollment and transported to the laboratory. We will measure the following:

1. Lymphocytes: circulating T lymphocytes (CD4^+^, CD8^+^), CD56^+^ natural killer cells, CD19^+^ B cells, and T regulatory cells.
2. Key cytokines and markers of inflammation: IFNγ, TNFα, IL-10, IL-17, IL-6, high-sensitivity C-reactive protein.
3. BBB integrity: MMP-9 (9).
4. Immunogenicity: HLA typing of hAEC and recipient.

## Results

The expected outcome from Aim 1 is the MTD to inform the dose to be used in a Phase 2 trial. A smaller vasogenic edema volume, a decrement in infarct expansion, and a decrement in fractional anisotropy volume with dose escalation will provide another assessment of safety and efficacy. It is expected that at the MTD, infarct expansion will be lower than at the preceding doses and in the control group. The immunological assays should parallel our findings in animal experiments (e.g., dampening of the pro-inflammatory cytokines). The findings of the study will be submitted for publication in peer-reviewed journals. Following this, the application will be made to the National Health Medical Research Council to obtain funding for Phase 2 trial.

## Discussion

If successful, stem cell therapy in the acute phase of ischemic stroke will be transformative for patients who miss out on clot-busting drugs or clot-retrieval therapies, in reducing disability and death. In line with recommendation by the STEPS3 experts, we have avoided including patients who have received TPA or ECR as these therapies would confound the effect of hAECs (8). Apart from allogenic multipotent adult progenitor cells, hAEC is the only other stem cell tested at present in the first 48 h (1, 7). This therapy is attractive as hAECs are readily sourced from healthy term placentae, typically, for logistical ease, after elective cesarean section. There are about 45,000 term elective cesarean section births in Australia alone each year, after which the placenta is discarded. Each term placenta can provide 200–300 million hAECs, sufficient for one stroke patient (70–80 kg) at a dose of 4 million cells per kg. At higher cell doses, pooled cells from two to three placentae would be required for each adult patient. Unlike other forms of stem cell therapy, such as immortalized human neural stem cells derived from 12-week-old human fetuses (18), there are no significant ethical concerns with the collection and therapeutic use of hAECs (25). The risk of tumorigenesis with hAECs is also extremely low due to their lack of expression of telomerase (26).

We have chosen the intravenous route of administration over direct intracerebral delivery as it is less invasive (does not require brain surgery). In our animal experiments, hAECs have action on other organs (such as spleen) affected by stroke. This modulation of the immune response does not require direct intracerebral injection.

From a safety aspect, we have also recently completed a Phase 1 safety trial of intravenously administered hAECs in six preterm infants with established bronchopulmonary dysplasia, using a dose of 1 million cells/kg (ACTRN 12614000174684). No severe adverse events were found to be associated with hAEC treatment. A Phase 1 trial of hAECs in adults with compensated cirrhosis is also underway in our institution (21) (ACTRN12616000437460).

## Conclusion

In summary, hAEC-based therapy is a highly innovative alternative to current reperfusion and neuroprotection strategies. In experimental models, hAECs appear to exert effects by modulating the immune response post stroke to limit the ongoing injury at the peri-infarct interface or the “inflammatory penumbra.” If ultimately proven to be successful in Phases 2 and 3, this therapy will have a greater proportion (80–90%) of eligible patients than current reperfusion therapies. It is anticipated that in future trials, we will test the additional benefit of hAECs with reperfusion therapies. This trial is registered with ANZCTRN (ACTRN12618000076279p).

## Ethics Statement

This trial has been approved by the Monash Health Human Research Ethics Committee.

## Author Contributions

Steering Committee: TP, HM, RL, EW, and CS. Writing group: TP, HM, RL, EW, and CS. Data Safety Monitoring Board: Geoffrey Donnan and Velandai Srikanth.

## Conflict of Interest Statement

The authors declare that the research was conducted in the absence of any commercial or financial relationships that could be construed as a potential conflict of interest.
